# Therapeutic Advances in Non-Small Cell Lung Cancer Harboring *EGFR* Exon 20 Insertion Mutations: From Molecular Biology to Targeted Therapy

**DOI:** 10.3390/ijms27093714

**Published:** 2026-04-22

**Authors:** Daniel Rosas, Jay Desai, Luis Raez

**Affiliations:** 1 Memorial Cancer Institute, Memorial Healthcare System, Herbert Wertheim College of Medicine, Florida International University, Miami, FL 33199, USA; 2 Hebert Wertheim College of Medicine, Florida International University, Miami, FL 33199, USA

**Keywords:** *EGFR* exon 20 insertion, non-small cell lung cancer, targeted therapy, tyrosine kinase inhibitors, bispecific antibodies, molecular oncology

## Abstract

Epidermal growth factor receptor (*EGFR*) exon 20 insertion (ex20ins) mutations are the third most common *EGFR* mutation subtype in non-small cell lung cancer (NSCLC), accounting for approximately 4–12% of all *EGFR*-mutated cases. Unlike classical *EGFR* mutations, ex20ins mutations confer inherent resistance to first-, second- and third-generation *EGFR* tyrosine kinase inhibitors (TKIs) due to unique structural alterations that lock the αC-helix in an active orientation, creating steric hindrance within the drug-binding pocket. Until recently, platinum-based chemotherapy remained the standard first-line treatment, with objective response rates (ORR) of 19–47% and a median progression-free survival (PFS) of 6–7 months. Over the past five years, the therapeutic landscape has shifted, driven by the development of selective inhibitors and bispecific antibodies. Amivantamab, a bispecific *EGFR*–mesenchymal–epithelial transition factor (*MET*) antibody combined with chemotherapy, demonstrated superior efficacy in the PAPILLON trial, with an ORR of 73% and a median PFS of 11.4 months in the first-line setting. Sunvozertinib, an oral, selective *EGFR* inhibitor, received U.S. Food and Drug Administration (FDA) accelerated approval in 2025, with an ORR of 46% and a median duration of response (DOR) of 11.1 months in platinum-pretreated patients. Emerging therapies, including zipalertinib and furmonertinib, have shown promising results in early-phase trials, with zipalertinib demonstrating activity in patients pretreated with amivantamab (ORR 31.5%) and furmonertinib achieving remarkable responses in treatment-naive patients (ORR 78.6% at 240 mg). This comprehensive review analyzes the molecular biology, structural mechanisms, current therapeutic options, and novel investigational agents for *EGFR* ex20ins-mutated NSCLC.

## 1. Introduction

Non-small cell lung cancer (NSCLC) accounts for approximately 80–85% of all lung cancer cases and remains the leading cause of cancer-related mortality worldwide, with over 2.2 million new cases and 1.8 million deaths reported in 2020 [1]. The discovery of oncogenic driver mutations in the epidermal growth factor receptor (*EGFR*) gene two decades ago revolutionized the treatment landscape for NSCLC, leading to targeted therapies that significantly improved survival outcomes for affected patients. *EGFR* mutations are among the most common molecular alterations in NSCLC, with prevalence ranging from 40 to 50% in Asian populations to 10–30% in Caucasian populations [2].

The majority of *EGFR* mutations—approximately 85–90%—are classical activating mutations, including exon 19 deletions and the exon 21 (L858R) point mutation [3]. These classical mutations confer exquisite sensitivity to *EGFR* tyrosine kinase inhibitors (TKIs), with objective response rates (ORRs) exceeding 70% and median progression-free survival (PFS) ranging from 9 to 18 months across first- through third-generation TKIs. However, approximately 10–15% of *EGFR*-mutant patients harbor less common *EGFR* mutations that show variable responses to approved *EGFR*-TKIs, posing significant therapeutic challenges.

Among uncommon *EGFR* mutations, exon 20 insertions (ex20ins) are the third most frequent subtype, after exon 19 deletions and L858R mutations. *EGFR* ex20ins accounts for 4–12% of all *EGFR*-mutated NSCLCs, with an estimated prevalence of 0.4–1.0% in the general NSCLC population and 6.1% among *EGFR*-positive cases [4,5]. Despite their lower frequency compared with classical mutations, *EGFR* ex20ins affect a clinically significant patient population—estimated at approximately 24,000 patients annually in the United States, Europe, and Japan combined—comparable in size to other genotype-defined NSCLC subsets, such as *ROS1* rearrangements or *BRAF* mutations [6].

The clinical characteristics of NSCLC patients with *EGFR* ex20ins mutations resemble those of patients with classical *EGFR* mutations, with enrichment for women (47–67%), Asian populations, never-smokers (56–67%), and adenocarcinoma histology [7]. However, patients with *EGFR* ex20ins mutations have historically had significantly worse outcomes than those with classical *EGFR* mutations, with median overall survival (OS) ranging from 11 to 24 months, compared with approximately 38.6 months in classical *EGFR*-mutant NSCLC [8]. This disparity stems primarily from the inherent resistance of *EGFR* ex20ins mutations to first-, second- and third-generation *EGFR*-TKIs, which yield ORRs of only 0–29% and median PFS of less than 3 months in this population. Consequently, platinum-based chemotherapy remained the standard first-line treatment for *EGFR* ex20ins-mutated NSCLC until recently [9].

Over the past five years, there has been remarkable progress in understanding the molecular mechanisms underlying *EGFR* ex20ins-mediated resistance and in developing novel therapeutic agents to target these mutations. Structural characterization of *EGFR* ex20ins mutations revealed that insertions in the αC-helix and adjacent loop region create a wedge that locks the receptor in an active conformation, resulting in steric hindrance that prevents binding of conventional *EGFR*-TKIs while leaving the ATP-binding pocket largely intact [10]. This mechanistic understanding has guided the rational design of mutation-selective inhibitors with improved activity against *EGFR* ex20ins mutations.

Recent approvals of amivantamab, a bispecific *EGFR*-*MET* antibody, and sunvozertinib, an oral selective *EGFR* inhibitor, have transformed the treatment paradigm for *EGFR* ex20ins-mutated NSCLC. Additionally, several investigational agents, including zipalertinib and furmonertinib, are being evaluated in clinical trials, offering hope for expanding therapeutic options for this previously underserved patient population. The emergence of combination strategies, such as amivantamab plus chemotherapy in the first-line setting, has demonstrated unprecedented efficacy, with median PFS exceeding 11 months [11].

**Figure 1:**
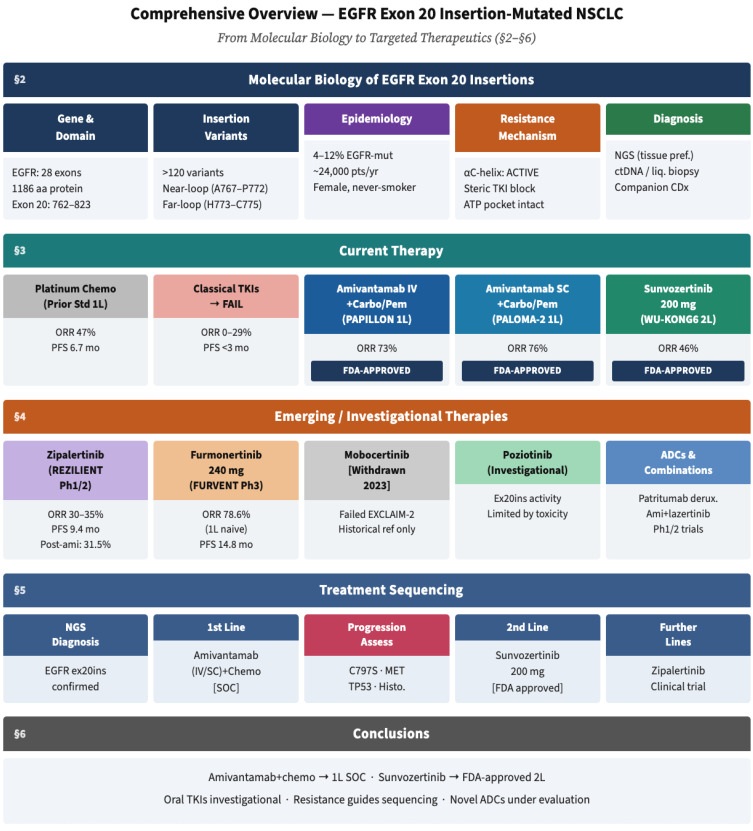
Comprehensive schematic overview of the manuscript. The figure summarizes the molecular biology of *EGFR* exon 20 insertion (ex20ins) mutations (§2), the structural basis of resistance to classical TKIs and subsequent development of targeted agents (§3), investigational therapies in active clinical trials (§4), evidence-based treatment sequencing (§5), and conclusions and future research priorities (§6). ORR, objective response rate; PFS, progression-free survival; SOC, standard of care; inv., investigational; 1L/2L, first-line/second-line.

A visual overview of the manuscript structure is provided in Figure 1. This comprehensive review provides an up-to-date synthesis of the current understanding of *EGFR* ex20ins mutations in NSCLC, covering molecular biology, structural mechanisms of resistance, established and emerging therapeutic options, and future research directions. We incorporate the latest data from major international oncology conferences, including ASCO 2025, ESMO 2025, and WCLC 2025, to offer clinicians and researchers evidence-based guidance for managing this challenging NSCLC subset.

## 2. Role of *EGFR* Exon 20 Insertions in Lung Cancer: Biology, Genetics, and Targetable Alterations

*EGFR* exon 20 insertion mutations encompass a highly heterogeneous group of molecular alterations. Table 1 provides an overview of the most clinically relevant variants, categorized by insertion type and approximate frequency, along with their established structural effects on the αC-helix region. These structural differences have direct therapeutic implications: near-loop insertions (A767–P772) generally confer broader drug sensitivity than far-loop insertions (H773–C775), while helical αC-helix insertions (e.g., A763_Y764insFQEA) exhibit atypical sensitivity to classical first-generation *EGFR*-TKIs. This molecular heterogeneity underscores the importance of precise genotyping by next-generation sequencing (NGS) rather than hotspot PCR assays for all patients with *EGFR*-mutated NSCLC.

### 2.1. Structure and Function of the EGFR Gene

The epidermal growth factor receptor (*EGFR*) is a transmembrane glycoprotein in the *ErbB* family of receptor tyrosine kinases, which also includes human epidermal growth factor receptor 2 (*HER2*/*ErbB*2), *HER3*/*ErbB*3, and *HER4*/*ErbB*4 [12]. *EGFR* comprises 28 exons that encode a 1186-amino acid protein with three functional domains: an extracellular ligand-binding domain, a single transmembrane domain, and an intracellular tyrosine kinase domain [13]. Upon ligand binding (e.g., epidermal growth factor or transforming growth factor-α), *EGFR* undergoes conformational changes that promote homodimerization or heterodimerization with other *ErbB* family members, resulting in autophosphorylation of tyrosine residues within the kinase domain [14].

*EGFR* activation initiates downstream signaling cascades, primarily through the *RAS-RAF-MEK-ERK* (MAPK) and *PI3K-AKT-mTOR* pathways, which regulate critical cellular processes, including proliferation, survival, differentiation, angiogenesis, and metastasis [15,16]. Under normal physiological conditions, *EGFR* signaling is tightly regulated and ligand-dependent; however, activating mutations cause constitutive, ligand-independent receptor activation that drives oncogenesis.

**Figure 2:**
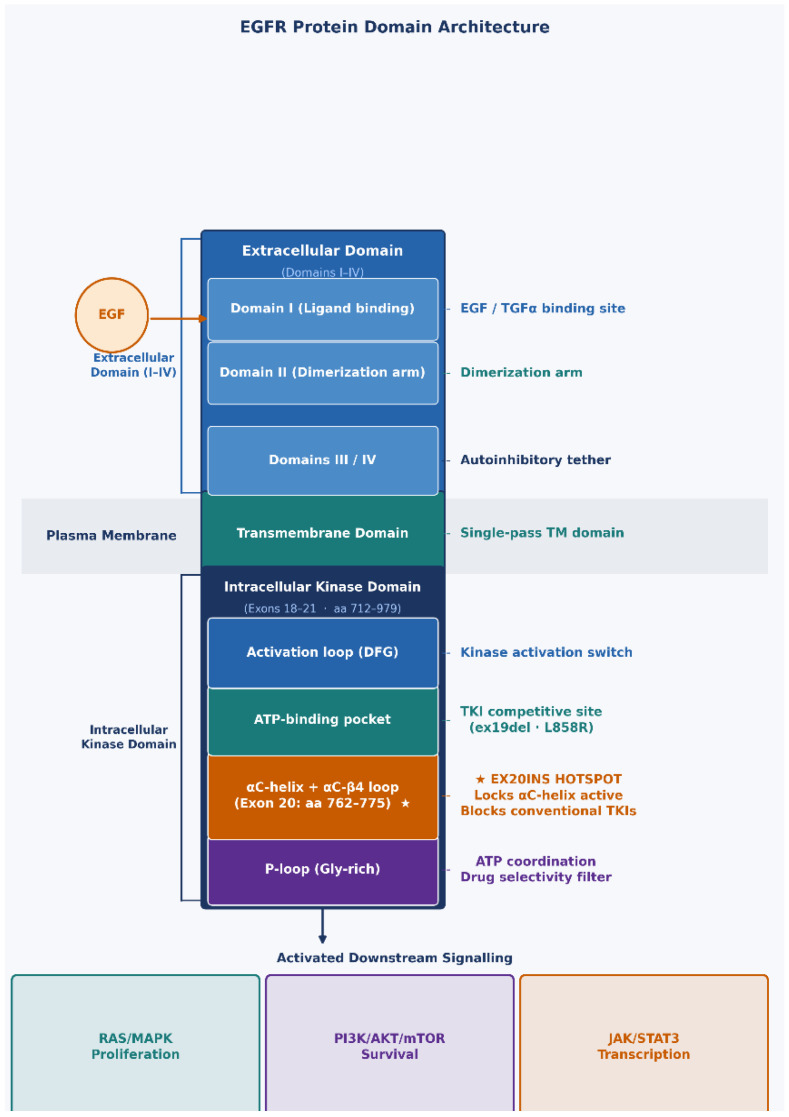
Schematic representation of the *EGFR* protein domain architecture and functional regions. The receptor consists of an extracellular ligand-binding domain (Domains I–IV), a single-pass transmembrane domain, and an intracellular tyrosine kinase domain encoded by exons 18–21 (amino acids 712–979). Key kinase sub-regions are shown: the P-loop (Gly-rich), ATP-binding pocket (hinge region), αC-helix + αC-β4 loop (Exon 20, aa 762–775; orange box), and the activation loop (A-loop). Exon 20 is the primary hotspot for insertion mutations. Downstream RAS/MAPK, PI3K/AKT/mTOR, and JAK/STAT3 signaling cascades activated by constitutively active *EGFR* are indicated on the left. EGF, epidermal growth factor; TGF-α, transforming growth factor-alpha.

Mutations in the *EGFR* gene predominantly occur in exons 18–21, which encode the tyrosine kinase domain spanning amino acids 712–979 [17]. The kinase domain contains several critical structural elements, including the phosphate-binding loop (P-loop), the αC-helix, the ATP-binding pocket, the activation loop, and the catalytic loop. Exon 20 encodes amino acids E762–K823, encompassing two functionally important regions: the αC-helix regulatory domain (E762–M766) and the adjacent αC-β4 loop (A767–C775) [18] (Figure 2).

### 2.2. Molecular Characteristics of EGFR Exon 20 Insertions

*EGFR* exon 20 insertion mutations are in-frame insertions or duplications of 3–21 base pairs (equivalent to 1–7 codons) that occur predominantly between codons 762 and 775 [19]. These insertions add one to seven amino acids within the αC-helix or the immediately adjacent loop region. Unlike classical *EGFR* mutations, ex20ins mutations exhibit remarkable molecular heterogeneity, with more than 120 distinct variants reported in the literature (Table 1) [20].

The two most common *EGFR* ex20ins variants—V769_D770insASV and D770_N771insSVD—account for approximately 40% of all ex20ins cases [21]. Other frequently observed insertions include A767_V769dupASV, H773_V774insNPH, D770_N771insG, and S768_D770dup. In a comprehensive analysis of 1500 lung adenocarcinomas, *EGFR* ex20ins mutations accounted for 9% of all *EGFR*-mutated tumors, with 13 distinct insertion types identified at the amino acid level. Subsequent studies using next-generation sequencing (NGS) have revealed even greater diversity, confirming that ex20ins mutations constitute the most heterogeneous family of activating *EGFR* mutations.

The location of insertions within exon 20 has important functional and therapeutic implications. Insertions can be categorized into three major groups based on their position: (1) near-loop insertions (A767-P772), occurring in the loop immediately adjacent to the αC-helix; (2) far-loop insertions (H773-C775), occurring in the more distal portion of the loop; and (3) helical insertions (approximately E762-Y764), occurring within the αC-helix itself [22]. Studies have shown that near-loop insertions are generally more sensitive to certain TKIs than far-loop insertions, likely due to subtle differences in drug-binding pocket geometry.

Notably, a small subset of exon 20 insertions within the αC-helix, particularly A763_Y764insFQEA, exhibits atypical behavior and sensitivity to first-generation *EGFR*-TKIs such as erlotinib. This variant, which accounts for less than 5% of ex20ins cases, appears to act through a distinct mechanism, with in vitro studies showing erlotinib IC_50_ values of less than 0.1 μM. Clinical data confirm that patients harboring this specific insertion can achieve tumor regression or stable disease with erlotinib, underscoring the importance of precise molecular characterization.

### 2.3. Molecular Mechanisms of Oncogenic Activation

*EGFR* ex20ins mutations drive oncogenesis by constitutively activating the receptor tyrosine kinase in a ligand-independent manner [23]. The activation mechanism differs fundamentally from that of classical *EGFR* mutations. Classical mutations, such as exon 19 deletions, destabilize the inactive conformation of *EGFR*, shifting the equilibrium toward the active state. In contrast, ex20ins mutations stabilize the active conformation by introducing structural constraints that prevent the receptor from adopting the inactive state.

**Figure 3:**
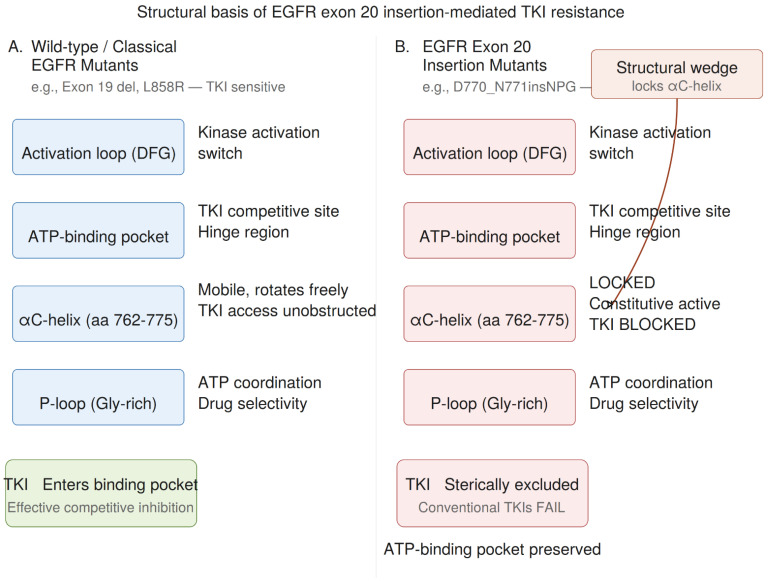
Structural basis of *EGFR* exon 20 insertion mutation–mediated resistance to tyrosine kinase inhibitors (TKIs). (**A**) In wild-type *EGFR* and classical activating mutants (e.g., exon 19 deletions, L858R), the αC-helix is mobile and the ATP-binding pocket is fully accessible to TKIs. (**B**) In *EGFR* exon 20 insertion mutants (e.g., D770_N771insNPG), inserted residues form a structural wedge at the C-terminus of the αC-helix, locking it in the active inward orientation. This creates a steric hindrance that prevents binding of conventional TKIs while leaving ATP affinity intact (Km ~10–30 μM). The differential explains the inherent resistance of ex20ins mutations to first-, second- and third-generation *EGFR*-TKIs. DFG, Asp-Phe-Gly motif; TKI, tyrosine kinase inhibitor.

Crystal structure analysis of *EGFR* harboring ex20ins mutations (e.g., D770_N771insNPG) revealed that the inserted amino acids form a wedge at the C-terminal end of the αC-helix (Figure 3). This wedge pushes the αC-helix into an inward, active orientation and prevents its rotation to the outward, inactive position that normally occurs in the absence of ligand binding. The αC-helix is a key regulatory element that must rotate inward to stabilize the active kinase conformation and facilitate dimerization-competent *EGFR*. By locking the αC-helix in the active position, ex20ins mutations ensure persistent receptor activation and constitutive downstream signaling through the RAS-RAF-MEK-ERK and PI3K-AKT-mTOR pathways.

Molecular dynamics simulations have provided additional insights into the conformational dynamics of *EGFR* ex20ins mutants [24]. These studies show that near-loop insertions (e.g., S768dupSVD) adopt multiple conformational states with lower transition-state energy barriers, potentially increasing flexibility in the drug-binding region. In contrast, far-loop insertions (e.g., H773insNPH) adopt fewer conformational states with higher transition-state energies, suggesting a more rigid structure that may further limit drug binding. These biophysical differences likely contribute to the observed heterogeneity in drug sensitivity among ex20ins variants.

Biochemical characterization of purified *EGFR* kinase domains harboring common ex20 insertions has shown that these variants exhibit intrinsic kinase activity comparable to wild-type *EGFR* in the active conformation [25]. However, unlike wild-type *EGFR*, which requires ligand-induced dimerization and conformational changes for activation, ex20ins mutants maintain constitutive activity in the absence of ligand. This constitutive activation drives persistent phosphorylation of downstream signaling molecules, creating a state of “oncogene addiction” that renders tumor cells dependent on continuous *EGFR* signaling for survival.

### 2.4. Structural Basis for Resistance to Conventional EGFR-TKIs

**Figure 4:**
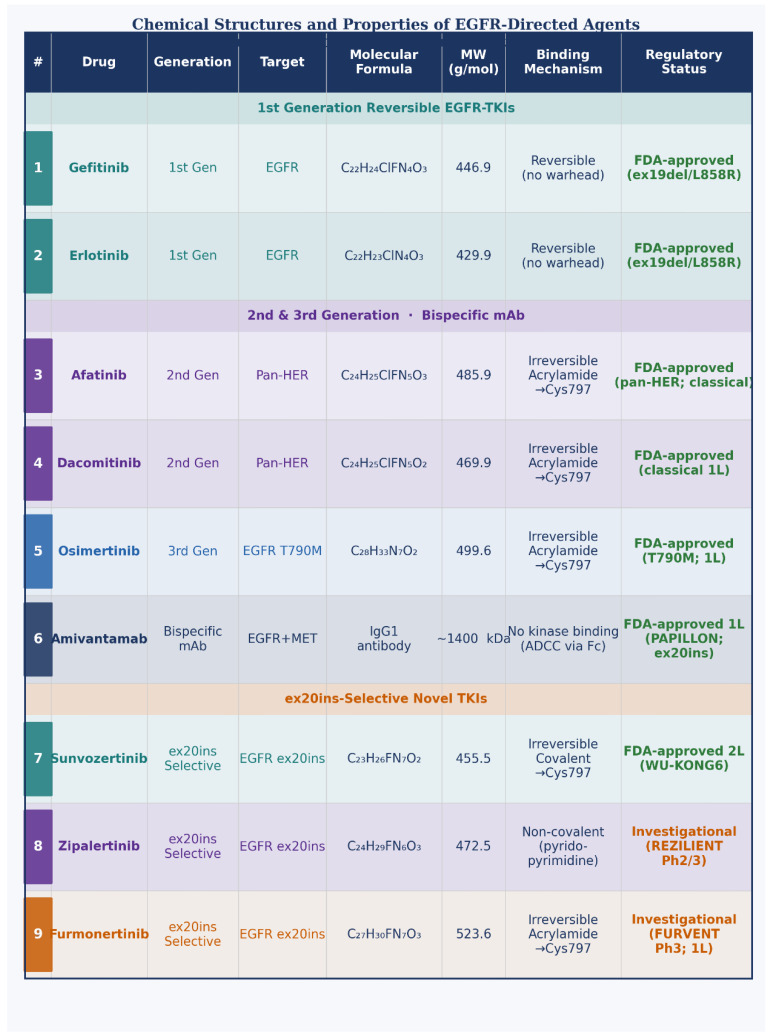
Schematic chemical structures of key *EGFR*-directed agents, numbered (1–9) by drug class and generation. Drugs 1 (gefitinib) and 2 (erlotinib) are first-generation reversible *EGFR*-TKIs. Drugs 3 (afatinib) and 4 (dacomitinib) are second-generation irreversible pan-HER TKIs with acrylamide warheads forming covalent bonds at Cys797. Drug 5 (osimertinib) is a third-generation T790M-selective covalent *EGFR*-TKI. Drug 6 (amivantamab) is a bispecific IgG1 monoclonal antibody that targets extracellular domains of *EGFR* and *MET*. Drugs 7 (sunvozertinib), 8 (zipalertinib), and 9 (furmonertinib) are novel exon 20 insertion-selective oral TKIs with compact scaffolds designed to fit the sterically constrained drug-binding pocket of *EGFR* ex20ins mutants while sparing wild-type *EGFR*. Structural representations are schematic and highlight core ring systems, heteroatoms, functional groups, and covalent warheads. TKI, tyrosine kinase inhibitor; mAb, monoclonal antibody; inv., investigational; FDA, U.S. Food and Drug Administration.

The chemical structures and binding mechanisms of all *EGFR*-directed agents discussed in this review are illustrated in Figure 4. The inherent resistance of *EGFR* ex20ins mutations to first-, second- and third-generation *EGFR*-TKIs stems from structural alterations that disrupt drug binding while preserving ATP binding [26]. Early hypotheses suggested that ex20ins mutations might directly occlude the ATP-binding pocket; however, crystallographic studies revealed that the insertion leaves the ATP-binding pocket largely unaltered. Instead, resistance arises from more subtle changes in pocket geometry and dynamics.

Kinetic analysis of *EGFR* ex20ins mutants shows that these variants retain ATP affinity comparable to wild-type *EGFR* (Km for ATP approximately 10–30 μM), whereas their affinity for first- and second-generation TKIs is markedly reduced [27]. For example, the IC_50_ of gefitinib for *EGFR* harboring the D770_N771insNPG insertion exceeds 2 μM, compared with less than 0.01 μM for classical activating mutations. This differential affinity arises because ex20ins mutations do not substantially alter ATP binding but create steric constraints that impede TKI binding.

The mechanism underlying this selective drug resistance involves insertion-induced conformational changes in the αC-helix and adjacent loop, which reduce the size and alter the shape of the drug-binding pocket [28]. Specifically, the insertion shifts the αC-helix and P-loop inward, creating steric hindrance that prevents optimal positioning of bulky TKI molecules. First- and second-generation *EGFR*-TKIs, such as gefitinib, erlotinib, and afatinib, were designed to inhibit classical *EGFR* mutants and wild-type *EGFR*, which have larger, more accessible drug-binding pockets. These agents cannot accommodate the constrained geometry imposed by ex20ins mutations, resulting in reduced binding affinity and loss of clinical efficacy.

Third-generation *EGFR*-TKIs, including osimertinib, were specifically developed to overcome T790M-mediated resistance in classical *EGFR*-mutant NSCLC by selectively inhibiting T790M-containing *EGFR* while sparing wild-type receptors [29]. However, osimertinib and other third-generation agents also show minimal activity against *EGFR* ex20ins mutations, with clinical response rates below 10%. This limited efficacy reflects that third-generation TKIs were optimized for the T790M gatekeeper mutation rather than the structural constraints imposed by ex20ins insertions.

Notably, a subset of second-generation pan-HER inhibitors, including afatinib, dacomitinib, and neratinib, has shown variable activity against certain *EGFR* ex20ins variants in preclinical models [30]. A comprehensive study of 60 ex20ins variants in engineered Ba/F3 cell lines found that insertions introducing glycine at position 770 (e.g., D770_N771insG) were uniquely sensitive to dacomitinib, with IC_50_ values in the nanomolar range. These findings suggest that the specific amino acid sequence and insertion site influence drug sensitivity, although clinical responses to second-generation pan-HER inhibitors remain limited in most patients.

### 2.5. Co-Occurring Genomic Alterations and Tumor Biology

*EGFR* ex20ins mutations are generally mutually exclusive with other major oncogenic drivers in NSCLC, including *KRAS* mutations, *ALK* rearrangements, and *ROS1* fusions. In a study of 1556 NSCLC patients with *EGFR* mutations, only 0.7% (11 of 1556) harbored concurrent *KRAS* mutations, and these were almost exclusively found in ex20ins or other uncommon *EGFR* mutation cases. This mutual exclusivity supports the concept that *EGFR* ex20ins mutations function as primary oncogenic drivers sufficient to initiate and maintain tumorigenesis.

Comprehensive genomic profiling has revealed that *EGFR* ex20ins-mutated tumors frequently harbor co-occurring genomic alterations in tumor suppressor genes and in genes involved in cell cycle regulation, DNA damage response, and chromatin modification [31]. In a cohort of 104 patients with *EGFR* ex20ins mutations, the most common co-occurring alterations were *TP53* mutations (45–60%), loss of *CDKN2A*/B (10–15%), and *MDM2* amplifications (5–10%). Less frequent co-occurring alterations included mutations or amplifications in PIK3CA, PTEN, RB1, and chromatin remodeling genes such as ARID1A and KMT2C.

The presence of specific co-occurring alterations may influence clinical outcomes and treatment responses. For instance, concurrent *TP53* mutations in *EGFR*-mutant NSCLC have been associated with more aggressive disease biology, higher rates of brain metastases, and, in some studies, reduced benefit from *EGFR*-TKI therapy [32]. Similarly, alterations in DNA damage response pathways (e.g., *ATM*, *ATR*, *BRCA1*/2) may affect sensitivity to platinum-based chemotherapy and could represent targets for combination therapeutic strategies.

The tumor microenvironment and immune landscape of *EGFR* ex20ins-mutated NSCLC differ from those of *EGFR* wild-type tumors. *EGFR* ex20ins-mutated tumors typically have a low tumor mutational burden (TMB), with median values of 3–4 mutations per megabase, similar to classical *EGFR* mutations and significantly lower than in *EGFR* wild-type tumors (8–10 mutations per megabase) [33]. This low TMB correlates with the limited efficacy of immune checkpoint inhibitors (ICIs) in unselected *EGFR*-mutant populations. However, emerging data suggest that *EGFR* ex20ins mutations may respond better to ICIs than classical *EGFR* mutations, with a reported ORR of 25% and a median PFS of 2.9 months in a series of 36 ex20ins patients, compared with 0% ORR and a median PFS of 1.9 months in 38 patients with classical mutations. This differential response warrants further investigation and may relate to distinct immunologic features of ex20ins-mutated tumors.

The clinical efficacy and key safety data for all currently available and emerging *EGFR* exon 20 insertion-directed therapies are summarized in Table 2. This comparison encompasses the established amivantamab-based regimens, the FDA-approved second-line agent sunvozertinib, and the investigational agents zipalertinib and furmonertinib that are discussed in detail in the following sections. Because these data derive from trials with different eligibility criteria, prior treatment requirements, and response assessment methodologies, cross-trial comparisons must be interpreted with caution. Nonetheless, the table provides a practical framework for understanding the relative efficacy and tolerability profiles that inform treatment sequencing decisions in clinical practice.

### 2.6. Comparison with HER2 Exon 20 Insertions

Human epidermal growth factor receptor 2 (*HER2*) is a close homolog of *EGFR* within the *ErbB* receptor tyrosine kinase family, and structurally analogous exon 20 insertion mutations occur in *HER2* in approximately 2% of NSCLC patients. HER2 ex20ins mutations account for over 90% of all HER2 mutations in NSCLC and share structural and functional similarities with *EGFR* ex20ins mutations.

The most common *HER2* ex20ins mutation is A775_G776insYVMA, which accounts for most *HER2* insertions [38]. Like *EGFR* ex20ins mutations, *HER2* ex20ins variants drive constitutive activation of the receptor tyrosine kinase through ligand-independent mechanisms. Both *EGFR* and *HER2* ex20ins mutations confer resistance to conventional TKIs targeting their respective receptors, necessitating the development of mutation-selective inhibitors.

Clinical characteristics of patients with *HER2* ex20ins-mutated NSCLC closely resemble those of patients with *EGFR* ex20ins mutations, with enrichment for adenocarcinoma histology, female sex, and never-smoking status [39]. Treatment outcomes with platinum-based chemotherapy are similar between *EGFR* and *HER2* ex20ins populations, with ORR of 13–35% and median PFS of 4–7 months. Notably, several *EGFR* ex20ins-selective inhibitors, including poziotinib and mobocertinib, the first developed for ex20ins, also demonstrate activity against *HER2* ex20ins mutations, reflecting the structural homology between these receptors.

Development of targeted therapies for *HER2* ex20ins has progressed in parallel with *EGFR* ex20ins-directed agents. In 2025, two *HER2*-directed TKIs—zongertinib and trastuzumab deruxtecan—received FDA approval for HER2-mutated NSCLC, with ORRs of 71–75% among patients with HER2 ex20ins [40]. These advances highlight the success of mutation-selective inhibitor development for exon 20 insertions across the *ErbB* receptor family.

### 2.7. Detection and Molecular Diagnosis

Accurate detection and characterization of *EGFR* ex20ins mutations are essential for guiding treatment decisions and clinical trial enrollment. The molecular heterogeneity of ex20ins mutations, with over 120 distinct variants, poses unique diagnostic challenges compared with classical *EGFR* mutations [41]. Traditional single-gene or hotspot PCR-based assays may miss certain ex20ins variants, particularly rare insertions or those occurring at atypical locations within exon 20.

Next-generation sequencing (NGS) has become the preferred diagnostic modality for comprehensive *detection* of *EGFR* mutations, including ex20ins variants. NGS enables simultaneous interrogation of all *EGFR* exons and can identify novel or rare insertions that targeted PCR assays may miss. In a comparative study of *EGFR* and *HER2* ex20ins detection, NGS identified ex20ins mutations in 53% of cases that were negative by real-time PCR, underscoring NGS’s superior sensitivity for detecting insertions. Contemporary guidelines recommend upfront NGS testing for all patients with advanced non-*EGFR* ex20ins mutations.

Both tissue-based and liquid biopsy approaches can detect *EGFR* mutations. Tissue biopsy remains the gold standard, and NGS analysis of tumor tissue provides the most comprehensive genomic information [42]. However, liquid biopsy using circulating tumor DNA (ctDNA) from peripheral blood has gained acceptance as a complementary or alternative diagnostic approach, particularly when tissue is insufficient or inaccessible.

Meta-analyses of liquid biopsy performance for *EGFR* mutation detection have shown that NGS-based ctDNA analysis achieves higher sensitivity (69%) than real-time quantitative PCR (qPCR; 56%) in treatment-naive NSCLC patients, while both methods maintain specificity above 89%. For *EGFR* ex20ins mutations specifically, liquid biopsy sensitivity may be lower than for classical mutations because of the lower allelic frequencies often observed with insertions. Nevertheless, liquid biopsy remains a valuable tool for molecular diagnosis, disease monitoring, and detection of resistance mechanisms.

Companion diagnostic assays have been developed and FDA-approved to guide treatment with targeted therapies for *EGFR* ex20ins. The Oncomine Dx Express Test received FDA approval as a companion diagnostic for sunvozertinib to detect *EGFR* ex20ins mutations in patients with NSCLC. Similarly, the Guardant360 CDx liquid biopsy assay and tissue-based NGS panels have been validated to identify patients eligible for amivantamab therapy.

Given the therapeutic implications of specific ex20ins variants, detailed molecular characterization should include not only the detection of an ex20ins mutation but also the precise identification of the insertion site and sequence. This information enables clinicians to distinguish near-loop from far-loop insertions, identify classical-like helical insertions (e.g., A763_Y764insFQEA), and potentially predict differential responses to available targeted therapies.

## 3. Current Therapy for *EGFR* Exon 20 Insertions in Lung Cancer

### 3.1. Platinum-Based Chemotherapy as First-Line Standard of Care

**Figure 5:**
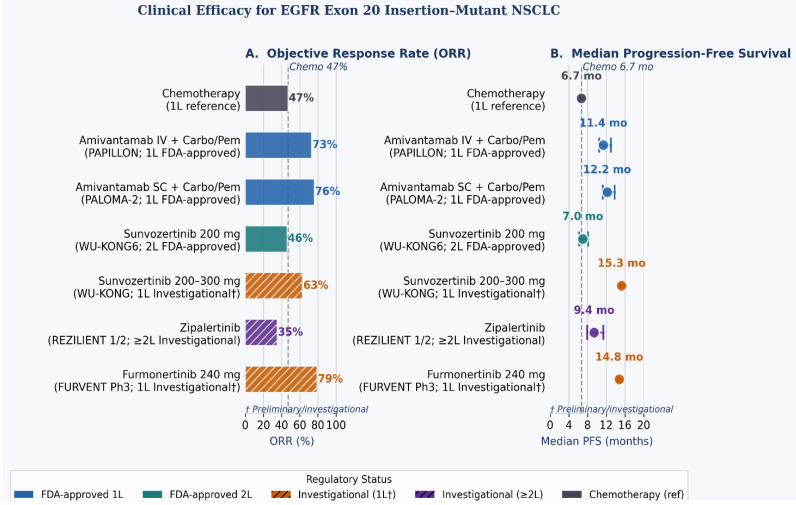
Clinical efficacy summary for established and emerging therapies in *EGFR* exon 20 insertion-mutant non-small cell lung cancer (NSCLC). (**A**) Objective response rates (ORR) by agent and treatment line. (**B**) Median progression-free survival (PFS) with 95% confidence interval bars where reported; Sunvozertinib is FDA-approved as second-line therapy for platinum-pretreated patients (WU-KONG6; ORR 46%, DOR 11.1 months); first-line data remain investigational. Furmonertinib 240 mg data are from the ongoing FURVENT Phase III trial and are investigational. Dashed vertical line denotes the platinum chemotherapy PFS benchmark (6.7 months, PAPILLON control arm). 1L, first-line; 2L, second-line; DOR, duration of response; SC, subcutaneous.

Until the recent approvals of targeted therapies for *EGFR* ex20ins mutations, platinum-based doublet chemotherapy was the standard first-line treatment for patients with advanced NSCLC harboring these mutations (Table 2) [43] (Figure 5). The rationale for chemotherapy stemmed from the inherent resistance of *EGFR* ex20ins mutations to first-, second- and third-generation *EGFR*-TKIs, which showed minimal clinical activity in this population, with ORR of 0–29% and median PFS of less than 3 months.

Real-world and retrospective studies have provided important data on the efficacy of platinum-based chemotherapy in *EGFR* ex20ins-mutated NSCLC. In a single-center retrospective analysis of 18 patients with advanced *EGFR* ex20ins NSCLC receiving first-line platinum-based chemotherapy, the ORR was 44%, and the median PFS was 7.1 months. The median OS in this cohort was 3.2 years, indicating that platinum-based chemotherapy can yield meaningful clinical outcomes in selected patients. These results compare favorably with historical data on platinum-based chemotherapy in classical *EGFR*-mutant NSCLC, where ORR ranges from 23 to 47%, and median PFS is 4.6–6.9 months in randomized trials.

A larger multicenter study of 104 Chinese patients with *EGFR* ex20ins-mutated NSCLC treated with first-line platinum-based chemotherapy reported an ORR of 19.2% and a median PFS of 6.4 months. Although the ORR was lower than in the single-center study, the PFS was consistent, supporting platinum-based chemotherapy as a reasonable treatment option in the absence of effective targeted therapies. The most commonly used regimen was carboplatin or cisplatin combined with pemetrexed, with or without bevacizumab.

The PAPILLON trial, which randomized patients to amivantamab plus chemotherapy or chemotherapy alone, provided prospective data on the efficacy of platinum-based chemotherapy in *EGFR* ex20ins-mutated NSCLC [44]. In the chemotherapy-alone arm (carboplatin AUC 5 plus pemetrexed 500 mg/m^2^ for 4 cycles, followed by pemetrexed maintenance), the median PFS was 6.7 months, and the ORR was 47%. These results from a randomized controlled trial confirm that platinum-pemetrexed chemotherapy yields a median PFS of approximately 6–7 months in patients with *EGFR* ex20ins NSCLC.

Despite these modest outcomes, platinum-based chemotherapy remained the only evidence-based first-line option for *EGFR* ex20ins-mutated NSCLC for many years. However, the advent of mutation-selective inhibitors and bispecific antibodies has transformed the treatment landscape.

### 3.2. Amivantamab: A Bispecific EGFR-MET Antibody

#### 3.2.1. Mechanism of Action and Preclinical Development

Amivantamab is a fully human IgG1 bispecific antibody that simultaneously targets the epidermal growth factor receptor (*EGFR*) and the mesenchymal–epithelial transition factor receptor (*MET*) tyrosine kinases. This dual-targeting design provides multiple antitumor mechanisms, distinguishing amivantamab from conventional small-molecule TKIs.

The therapeutic activity of amivantamab is mediated by three principal mechanisms. First, amivantamab blocks ligand binding to both *EGFR* and *MET*, thereby inhibiting ligand-induced receptor activation and downstream signaling. Second, amivantamab promotes receptor internalization and degradation, resulting in sustained downregulation of *EGFR* and *MET* surface expression. Third, and perhaps most importantly, amivantamab engages immune effector functions through its Fc domain, triggering antibody-dependent cellular cytotoxicity (ADCC) mediated by natural killer cells and antibody-dependent cellular phagocytosis (ADCP) by macrophages.

#### 3.2.2. Clinical Efficacy in *EGFR* Exon 20 Insertions

The pivotal CHRYSALIS study (NCT02609776), a multicenter Phase I trial, established the clinical activity of amivantamab in heavily pretreated patients with *EGFR* ex20ins-positive NSCLC [45]. In the Phase I portion, which included 81 patients who had progressed after platinum-based chemotherapy, amivantamab at the recommended dose of 1050 mg (for patients weighing <80 kg) or 1400 mg (for patients ≥80 kg), administered intravenously weekly for the first four weeks and then every two weeks thereafter, demonstrated an ORR of 40% (95% confidence interval [CI]: 29–51%) per independent central review. The median duration of response (DOR) was 11.1 months, the median PFS was 8.3 months, and the median OS was 22.8 months. These compelling results led to FDA accelerated approval of amivantamab in May 2021 for patients with locally advanced or metastatic NSCLC harboring *EGFR* exon 20 insertions whose disease progressed on or after platinum-based chemotherapy. 

The landmark PAPILLON trial (NCT04538664) is an open-label, randomized Phase III study comparing amivantamab plus carboplatin-pemetrexed chemotherapy with chemotherapy alone in treatment-naive patients with *EGFR* ex20ins-mutated NSCLC. Results presented at ESMO 2024 showed a statistically significant and clinically meaningful improvement in PFS with the combination: median PFS was 11.4 months with amivantamab plus chemotherapy versus 6.7 months with chemotherapy alone (hazard ratio [HR] 0.40; 95% confidence interval [CI]: 0.30–0.53; *p* < 0.0001). The ORR was also higher in the combination arm (73% vs. 47%), with a confirmed clinical benefit rate of 94% by investigator assessment. Median duration of response (DOR) exceeded 10 months, and at a median follow-up of 10.4 months, median overall survival (OS) was not reached. These results established amivantamab plus chemotherapy as a new standard of care for first-line treatment of *EGFR* ex20ins-mutated NSCLC.

#### 3.2.3. Subcutaneous Formulation

The Phase III PALOMA-3 trial (NCT05388669) established the pharmacokinetic (PK) noninferiority and superior safety of subcutaneous (SC) amivantamab compared with intravenous (IV) amivantamab in patients with *EGFR*-mutated advanced NSCLC. The study showed that SC administration achieved plasma drug exposure comparable to IV administration, with significantly lower rates of infusion- or administration-related reactions (IRRs/ARRs). Based on PALOMA-3 results, the FDA approved the subcutaneous formulation of amivantamab for all existing amivantamab indications in December 2024, offering patients and clinicians a more convenient administration option with a substantially improved tolerability profile.

The PALOMA-2 trial evaluated SC amivantamab plus chemotherapy as first-line treatment for advanced NSCLC harboring *EGFR* ex20ins mutations. Cohort 2 enrolled 66 patients who received SC amivantamab (1680 mg, co-formulated with hyaluronidase) on cycle 1 days 1, 8, and 15, then every 3 weeks starting in cycle 2, in combination with carboplatin and pemetrexed. The study met its primary endpoint, with an ORR of 76% (95% CI 64–86) by investigator assessment and 77% (95% CI 65–87) by independent central review. Median DOR was 10.6 months. At a median follow-up of 10.4 months, median PFS was 12.2 months, and median OS was not reached. Importantly, the SC formulation demonstrated a markedly improved safety profile compared with IV administration, particularly for administration-related reactions. ARRs occurred in only 6% of patients receiving SC amivantamab in PALOMA-2, a 7-fold reduction compared with the 42% IRR rate with IV amivantamab in PAPILLON. All ARRs were Grade 1–2 and occurred during the first injection. PK analysis confirmed that SC amivantamab achieved mean plasma concentrations comparable to IV administration, with a mean amivantamab concentration of 439 μg/mL on cycle 2 day 1, consistent with historical IV data. The SC formulation therefore provides PK and efficacy equivalent to IV administration, with superior convenience and tolerability [46].

#### 3.2.4. Safety Profile

The most common adverse events with amivantamab are infusion/administration-related reactions (IRRs/ARRs), occurring in approximately 60–65% of patients receiving IV administration, typically during the first infusion. To mitigate IRRs, amivantamab is administered with premedication, including antihistamines and corticosteroids, and the first IV infusion is split over two days. SC administration significantly reduces this incidence to 6%.

Other common toxicities with IV administration include rash (including acneiform dermatitis in 75–85% of patients), nail toxicity (paronychia in 25–35%), hypoalbuminemia, and peripheral edema. Interstitial lung disease/pneumonitis occurs in approximately 3% of patients and requires vigilant monitoring. Venous thromboembolism has been reported in 10–15% of patients, necessitating consideration of thromboprophylaxis, particularly when combined with chemotherapy. Most adverse events are manageable with dose modifications, supportive care, and dermatologic interventions. In PALOMA-2, treatment discontinuation due to AEs occurred in 12% of patients.

### 3.3. Sunvozertinib: An Oral Selective EGFR Inhibitor

#### 3.3.1. Development and Mechanism of Action

Sunvozertinib is an oral, next-generation, irreversible *EGFR* tyrosine kinase inhibitor designed to overcome the structural constraints imposed by *EGFR* ex20ins mutations. Unlike earlier-generation *EGFR*-TKIs developed for classical *EGFR* mutations, sunvozertinib was rationally designed to potently inhibit *EGFR* ex20ins mutants while maintaining selectivity for wild-type *EGFR*, thereby reducing dose-limiting toxicities such as rash and diarrhea that commonly occur with less selective *EGFR* inhibitors.

Preclinical studies demonstrated that sunvozertinib potently inhibits diverse *EGFR* ex20ins variants in engineered cell lines and patient-derived xenograft models. The selectivity of ex20ins-mutant *EGFR* over wild-type receptors provides a favorable therapeutic window, enabling effective target inhibition at doses with manageable toxicity. Additionally, sunvozertinib shows good brain penetration in preclinical models, suggesting potential activity against central nervous system (CNS) metastases.

Structurally, sunvozertinib incorporates design features that enable it to overcome the steric constraints imposed by ex20ins mutations. Although the crystal structures of sunvozertinib bound to *EGFR* ex20ins mutants have not been published, biochemical data confirm its potent and selective activity against multiple ex20ins variants.

#### 3.3.2. Clinical Efficacy: WU-KONG Trials

Clinical development of sunvozertinib included multiple Phase I and Phase II trials conducted in China and globally, collectively referred to as the WU-KONG program. The WU-KONG1 study, a Phase I/II trial in China, evaluated sunvozertinib in patients with locally advanced or metastatic NSCLC harboring *EGFR* ex20ins mutations. In the dose-escalation phase, sunvozertinib demonstrated a favorable safety profile, with no dose-limiting toxicities at 300 mg once daily, which was selected as the recommended Phase II dose.

In platinum-pretreated patients (WU-KONG1B), sunvozertinib at 300 mg once daily achieved an ORR of 61% and a median DOR exceeding 12 months. A dose-comparative analysis found that 200 mg daily had a more favorable safety profile while maintaining efficacy (ORR 46% at 200 mg vs. 58% at 300 mg).

The most recent data come from the WU-KONG6 trial, a Phase II study with extended follow-up. In platinum-pretreated patients, sunvozertinib achieved a confirmed ORR of 46% by independent review, a median DOR of 11.1 months, and a median PFS of 6–8 months. These results supported the FDA’s accelerated approval of sunvozertinib in July 2025 for patients with *EGFR* exon 20 insertions whose disease progressed on or after platinum-based chemotherapy.

In treatment-naive patients, preliminary data from the WU-KONG trials showed even more impressive activity, with an ORR of approximately 63% and a median PFS of 15+ months, suggesting that sunvozertinib may be particularly effective when used earlier in the treatment course.

#### 3.3.3. Safety and Tolerability

Sunvozertinib’s safety profile is notably milder than that of non-selective *EGFR* inhibitors, reflecting its selectivity for mutant over wild-type *EGFR*. The most common treatment-related adverse events include diarrhea (51%), rash (39%), stomatitis (28%), and increased alanine aminotransferase (26%), with most events of Grade 1–2. The rate of severe (Grade ≥ 3) rash was approximately 7%, significantly lower than that observed with afatinib or dacomitinib.

QT prolongation was observed in a small proportion of patients (<5%), necessitating electrocardiographic monitoring. Dose reductions or treatment interruptions were required in approximately 20% of patients [34]. Interstitial lung disease (ILD) occurred infrequently. These tolerability advantages, together with comparable or superior efficacy relative to other agents, make sunvozertinib an attractive option for both treatment-naive and previously treated patients.

### 3.4. Other Available Therapies

#### 3.4.1. Mobocertinib

Mobocertinib (TAK-788) is an oral, irreversible *EGFR* inhibitor developed specifically for *EGFR* exon 20 insertions. It received accelerated FDA approval in September 2021 based on the Phase I/II EXCLAIM study [47]. In platinum-pretreated patients, mobocertinib 160 mg once daily demonstrated an ORR of 28% and a median PFS of 7.3 months, with a median DOR of approximately 17.5 months [48].

However, the safety profile was challenging, with high rates of diarrhea (92%, Grade ≥ 3 in 22%), rash, nausea, and vomiting. The confirmatory Phase III EXCLAIM-2 trial, which compared mobocertinib with platinum-based chemotherapy in the first-line setting, failed to meet its primary endpoint of improved PFS. The sponsor subsequently withdrew the drug from the US market in 2023. Clinicians should remain aware of mobocertinib’s adverse event profile when evaluating patients who may have previously received this agent.

#### 3.4.2. Position of Third-Generation TKIs

For specific *EGFR* exon 20 insertion variants, third-generation TKIs may be considered. As noted earlier, certain near-C-helix insertions, particularly A763_Y764insFQEA, may respond to osimertinib or other third-generation agents due to their distinct structural properties. Case series have reported durable responses to osimertinib in patients harboring this variant, suggesting that detailed molecular characterization should guide treatment selection. However, these osimertinib-sensitive variants constitute only a small minority (approximately 5–10%) of all exon 20 insertions.

## 4. Emerging Therapies for *EGFR* Exon 20 Insertions

### 4.1. Zipalertinib: Next-Generation Selective Inhibitor

Zipalertinib (DZD0710, CLN-081, TAS6417) is an investigational, highly selective, irreversible *EGFR* TKI engineered to target *EGFR* exon 20 insertion mutations while preserving selectivity for wild-type *EGFR*. Developed through structure-guided medicinal chemistry, zipalertinib was designed to optimize binding within the constrained ATP-binding pocket of exon 20 insertion mutants.

Preclinical studies demonstrated potent, selective inhibition of diverse *EGFR* ex20ins variants in cell-based assays and tumor growth inhibition in patient-derived xenograft models. Notably, zipalertinib showed 10–100-fold selectivity for ex20ins-mutant *EGFR* over wild-type receptors in biochemical and cellular assays, suggesting an improved therapeutic window compared with earlier-generation TKIs.

The pivotal REZILIENT studies (Phase I/II trials) evaluated zipalertinib in heavily pretreated patients with *EGFR* exon 20 insertion-positive NSCLC. In the expanded Phase II cohorts of platinum-pretreated patients, zipalertinib demonstrated an overall ORR of approximately 30%, including CNS responses. Notably, responses varied by insertion location: an analysis showed an ORR of 42% in patients with “near-loop” insertions (closer to the C-helix) versus 22% in those with “far-loop” insertions, reflecting variable sensitivity by insertion location.

In a subset of patients who had previously received amivantamab, zipalertinib produced an ORR of approximately 31.5% (REZILIENT-2 cohort), suggesting potential use after antibody therapy and addressing the important question of sequencing after amivantamab resistance. Safety profiles are favorable, with rash and diarrhea as the most common adverse events; interstitial lung disease (ILD) has been reported rarely. Ongoing trials are now evaluating zipalertinib in first-line settings (REZILIENT-3, NCT05469113).

These results have generated enthusiasm that zipalertinib may become a standard option for exon 20-mutant NSCLC, particularly given its activity in both amivantamab-naive and amivantamab-pretreated patients, thereby offering sequential treatment options.

### 4.2. Furmonertinib: Next-Generation Irreversible EGFR TKI

Furmonertinib (HS-10296) is an irreversible *EGFR* TKI approved in China for *EGFR*-mutant NSCLC harboring sensitizing mutations [49]. Early phase data for exon 20 insertions are particularly encouraging. In a small cohort of patients treated at a higher dose (160 mg daily), furmonertinib achieved an ORR of 66.7% and a disease control rate (DCR) of 88.9%, with a median PFS of approximately 7.2 months [50]. Patients with brain metastases also responded, indicating good CNS penetration. The higher dose was well tolerated, with no Grade ≥3 adverse events reported.

These results prompted the Phase III FURVENT trial (NCT05019560), which evaluates furmonertinib (80 vs. 160 mg) versus standard chemotherapy in first-line *EGFR* exon 20 NSCLC. Interim presentations at WCLC 2024 and ASCO 2025 have shown ORRs of approximately 60–70% in untreated patients receiving furmonertinib at 240 mg (in a subsequent dose-optimization), far outperforming chemotherapy [35]. Importantly, furmonertinib appears active across insertion subtypes, with the high dose achieving an ORR of 78.6% in treatment-naive patients [36]. This remarkable efficacy has positioned furmonertinib as potentially the most active single agent identified to date for *EGFR* ex20ins NSCLC.

Ongoing studies are examining furmonertinib in combination with VEGF inhibitors (analogous to the FLORA trial with osimertinib in classical *EGFR* mutations) and its impact on acquired resistance mechanisms. If approved, furmonertinib could become another important oral targeted option for these patients, particularly valuable as a first-line monotherapy alternative to combination amivantamab-chemotherapy.

### 4.3. Other Investigational Approaches

#### 4.3.1. Poziotinib

Poziotinib showed modest activity (e.g., an overall ORR of approximately 32% and up to 46% for certain insertion subtypes), but significant adverse events limited its clinical utility. Its role remains investigational and has not gained widespread adoption compared with more selective agents in exon 20s. The U.S. FDA denied approval for poziotinib in late 2022 for previously treated non-small cell lung cancer (NSCLC) with *HER2* exon 20 insertion mutations, citing insufficient data from its Phase 2 trial (ZENITH20) and concerns about its benefit-to-risk profile relative to existing treatments such as trastuzumab deruxtecan, with many patients experiencing significant side effects.

#### 4.3.2. Antibody–Drug Conjugates

Clinical trials of antibody–drug conjugates (ADCs) targeting *EGFR* are also underway. Depatuxizumab mafodotin and other *EGFR*-directed ADCs deliver cytotoxic payloads to *EGFR*-expressing tumor cells via antibody-mediated delivery. Although early-phase data in unselected NSCLC populations have shown modest activity, the specific efficacy in *EGFR* exon 20 insertion-positive tumors remains to be fully characterized. These approaches represent an alternative mechanism-based strategy that does not rely on kinase inhibition and may therefore be less susceptible to the same resistance mechanisms as TKIs.

#### 4.3.3. Combination Strategies

Beyond the established amivantamab plus chemotherapy regimen, emerging combinations are under active investigation to further improve outcomes and delay resistance. These include amivantamab combined with *EGFR* TKIs such as lazertinib, and TKI plus antiangiogenic agent strategies analogous to the FLORA paradigm in classical *EGFR*-mutant NSCLC [37].

## 5. Treatment Sequencing and Clinical Decision-Making

The advent of multiple effective therapeutic options for *EGFR* ex20ins-mutated NSCLC has raised important questions about optimal treatment sequencing and patient selection. Current treatment paradigms continue to evolve as trial data and clinical experience emerge.

### 5.1. First-Line Treatment Strategies

**Figure 6:**
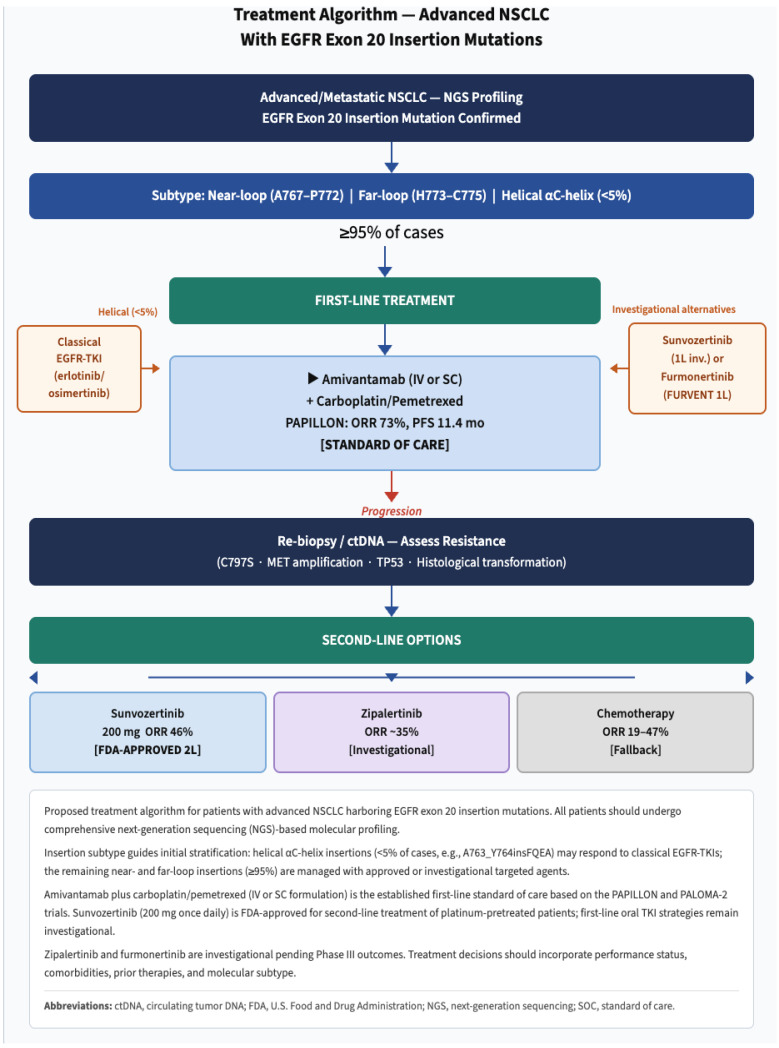
Proposed treatment algorithm for patients with advanced NSCLC harboring *EGFR* exon 20 insertion mutations. All patients should undergo comprehensive next-generation sequencing (NGS)-based molecular profiling. Insertion subtype guides initial stratification: helical αC-helix insertions (<5% of cases, e.g., A763_Y764insFQEA) may respond to classical *EGFR*-TKIs; the remaining near- and far-loop insertions (≥95%) are managed with approved or investigational targeted agents. Amivantamab plus carboplatin/pemetrexed (IV or SC formulation) is the established first-line standard of care based on the PAPILLON and PALOMA-2 trials. Sunvozertinib (200 mg once daily) is FDA-approved for second-line treatment of platinum-pretreated patients; first-line oral TKI strategies remain investigational. Zipalertinib and furmonertinib are investigational pending Phase III outcomes. Treatment decisions should incorporate performance status, comorbidities, prior therapies, and molecular subtype. ctDNA, circulating tumor DNA; FDA, U.S. Food and Drug Administration; NGS, next-generation sequencing; SOC, standard of care.

For treatment-naive patients with advanced NSCLC harboring *EGFR* ex20ins mutations, amivantamab plus carboplatin-pemetrexed (based on PAPILLON results) is a well-established first-line standard of care (Figure 6). This combination demonstrates superior PFS (11.4 months) and ORR (73%) compared with chemotherapy alone and has been adopted by major oncology guidelines.

However, emerging data from sunvozertinib trials in treatment-naive patients, showing an ORR of 63% and a median PFS of 15+ months, along with particularly exceptional results from furmonertinib at high doses (ORR 78.6%), raise the possibility that oral monotherapy may be a competitive alternative, particularly for its convenience and tolerability. The ongoing FURVENT trial comparing furmonertinib with chemotherapy will provide definitive prospective data comparing an oral TKI with standard chemotherapy in the first-line setting.

For patients with specific insertion variants (particularly near-C-helix insertions such as A763_Y764insFQEA), consideration of osimertinib or other third-generation TKIs, based on detailed molecular characterization, may be appropriate.

### 5.2. Second-Line and Beyond Treatment

For patients who progress on first-line chemotherapy or amivantamab-chemotherapy, the choice of second-line therapy depends on prior treatment exposure. Patients who received first-line chemotherapy alone are candidates for amivantamab monotherapy or sunvozertinib as second-line therapy. Patients who receive first-line amivantamab-chemotherapy have several options, including oral TKIs such as sunvozertinib, zipalertinib (which has demonstrated post-amivantamab activity), or furmonertinib.

For patients progressing on oral TKI monotherapy, options include switching to an alternative oral TKI, combining with chemotherapy, adding amivantamab if not previously received, or enrolling in clinical trials of novel agents.

### 5.3. Managing Resistance and Emerging Mechanisms

As patients receive these targeted therapies, acquired resistance will inevitably emerge. Understanding resistance mechanisms is crucial for guiding subsequent treatment selection and developing rational combination strategies [51]. Common acquired resistance mechanisms identified to date include on-target *EGFR* mutations (e.g., C797S in the context of covalent TKIs), *MET* amplification-mediated resistance to amivantamab, and off-target bypass pathway activation [52]. Prospective re-biopsy at progression and serial ctDNA monitoring are recommended to characterize resistance and inform subsequent therapy.

## 6. Conclusions and Future Directions

*EGFR* exon 20 insertion mutations define a biologically distinct subset of NSCLC that has historically been challenging to treat because of inherent resistance to conventional *EGFR* TKIs. Over the past five years, this once “undruggable” subgroup has rapidly evolved into a therapeutic success story. The bispecific antibody amivantamab and the novel *EGFR* inhibitors sunvozertinib, zipalertinib, and furmonertinib have each demonstrated clinically meaningful activity, with response rates and progression-free survival substantially exceeding those of historical chemotherapy.

## Figures and Tables

**Table 1:** Common *EGFR* exon 20 insertion variants in non-small cell lung cancer (NSCLC): mutation type, relative frequency, and structural effect on the αC-helix region. A comprehensive overview of the heterogeneity of *EGFR* exon 20 variants in NSCLC and preclinical activity to currently available treatments.

Common *EGFR* Exon 20 Mutations			
Mutation	Mutation Type	Relative Frequency	Structural Effect
V769_D770insASV	Insertion	Rare	Stabilizes C-helix
D770_N771insSVD	Insertion	Less Common	Causes steric hindrance in the drug-binding pocket
A767_V769dupASV	Duplication	Very Common	Stabilizes C-helix
H773_V774insNPH	Insertion	Very Common	Reduces autoinhibitory interactions of C-helix
D770_N771insG	Insertion	Less Common	Activates *EGFR* in a ligand-independent manner
S768_D770dup	Duplication	Very Common	Stabilizes active conformation and causes steric hindrance

**Table 2:** Clinical efficacy and key safety findings of currently available and emerging *EGFR* exon 20 insertion-directed therapies in NSCLC [11,22,34,35,36]. A comparison of objective response rates, progression-free survival, and adverse event profiles across approved and investigational agents.

Drug/Regimen	Key Trial	ORR	Median PFS	Grade 1–2 AEs	Grade 3–4 AEs
Amivantamab + Carboplatin/Pemetrexed	PAPILLON phase 3 (NCT04538664) [11]	73% (vs. 47% chemo alone) [11]	11.4 months (vs. 6.7 chemo alone) [11]	Rash, paronychia, infusion reactions [11]	Neutropenia, infusion reactions, stomatitis [11]
Sunvozertinib	WU-KONG1B phase 2 (NCT03974022) [34]	46% [34]	~6–8 months [34]	Diarrhea, rash, stomatitis [34]	Increase CPK levels, anemia, arrhythmia [34]
Zipalertinib	REZILIENT1 phase 1/2 (NCT04036682) [22]	35.2% [22]	9.4 months [22]	Paronychia, rash, diarrhea [22]	Anemia, pneumonitis, stomatitis [22]
Furmonertinib	FAVOUR phase 1b (NCT04858958) [35]; FURVENT phase 3 (NCT05607550) [36]	78.6% [37]	NR (phase 1b) [35]; phase 3 ongoing [36]	Diarrhea, anemia, transaminase evolution, rash [35]	QT prolongation, mouth ulceration, leukopenia [35]

Abbreviations: AE, adverse event; CPK, creatine phosphokinase; *EGFR*, epidermal growth factor receptor; NSCLC, non-small cell lung cancer; ORR, objective response rate; PFS, progression-free survival. Notes: Efficacy and safety data are extracted from the cited primary publications or conference materials. Cross-trial comparisons should be interpreted cautiously because of differences in eligibility criteria, prior lines of therapy (including prior exon20ins-directed agents), and assessment methodology.

## Data Availability

No new data were created or analyzed in this study. Data sharing is not applicable to this article.

## References

[1] Sung H., Ferlay J., Siegel R.L., Laversanne M., Soerjomataram I., Jemal A., Bray F. (2021). Global cancer statistics 2020: GLOBOCAN estimates of incidence and mortality worldwide for 36 cancers in 185 countries. CA Cancer J. Clin..

[2] Rosell R., Carcereny E., Gervais R., Vergnenegre A., Massuti B., Felip E., Palmero R., Garcia-Gomez R., Pallares C., Sanchez J.M. (2012). Erlotinib versus standard chemotherapy as first-line treatment for European patients with advanced *EGFR* mutation-positive non-small-cell lung cancer (EURTAC): A randomized open-label phase 3 trial. Lancet Oncol..

[3] Lynch T.J., Bell D.W., Sordella R., Gurubhagavatula S., Okimoto R.A., Brannigan B.W., Harris P.L., Haserlat S.M., Supko J.G., Haluska F.G. (2004). Activating mutations in the epidermal growth factor receptor underlying the responsiveness of non-small-cell lung cancer to gefitinib. N. Engl. J. Med..

[4] Arcila M.E., Nafa K., Chaft J.E., Rekhtman N., Lau C., Reva B.A., Zakowski M.F., Kris M.G., Ladanyi M. (2013). *EGFR* exon 20 insertion mutations in lung adenocarcinomas: A distinct subset of EGFR mutations that do not respond to EGFR tyrosine kinase inhibitors. Mol. Cancer Ther..

[5] Oxnard G.R., Lo P.C., Nishino M., Dahlberg S.E., Lindeman N.I., Butaney M., Jackman D.M., Johnson B.E., Janne P.A. (2013). Natural history and molecular characteristics of lung cancers harboring *EGFR* exon 20 insertions. J. Thorac. Oncol..

[6] Burnett H., Emich H., Carroll C., Stapleton N., Mahadevia P., Li T. (2021). Epidemiological and clinical burden of *EGFR* exon 20 insertion in advanced non-small cell lung cancer: A systematic literature review. PLoS ONE.

[7] Watanabe N., Horio Y., Fujiwara Y. (2022). Emerging therapies for non-small cell lung cancer harboring *EGFR* exon 20 insertion mutations: Narrative review. Ann. Transl. Med..

[8] Seo D., Lim J.H. (2024). Targeted therapies for *EGFR* exon 20 insertion mutation in non-small-cell lung cancer. Int. J. Mol. Sci..

[9] Christopoulos P. (2025). A new era for *EGFR* exon 20 insertion mutation targeting NSCLC. Chin. Clin. Oncol..

[10] Yasuda H., Park E., Yun C.H., Sng N.J., Lucena-Araujo A.R., Grber W.L., Neel D.S., Dagogo-Jack I., Rodig S.J., Muzikansky A. (2013). Structural, biochemical, and clinical characterization of *EGFR* exon 20 insertion mutations in lung cancer. Sci. Transl. Med..

[11] Zhou C., Tang K.-J., Cho B.C., Liu B., Paz-Ares L., Cheng S., Kitazono S., Thiagarajan M., Goldman J.W., Sabari J.K. (2023). Amivantamab plus chemotherapy in NSCLC with *EGFR* exon 20 insertions. N. Engl. J. Med..

[12] Hynes N.E., Lane H.A. (2005). ERBB receptors and cancer: The complexity of targeted inhibitors. Nat. Rev. Cancer.

[13] Lemmon M.A., Schlessinger J. (2010). Cell signaling by receptor tyrosine kinases. Cell.

[14] Yarden Y., Sliwkowski M.X. (2001). Untangling the *ErbB* signalling network. Nat. Rev. Mol. Cell Biol..

[15] Downward J. (2003). Targeting RAS signaling pathways in cancer therapy. Nat. Rev. Cancer.

[16] Vivanco I., Sawyers C.L. (2002). The phosphatidylinositol 3-kinase AKT pathway in human cancer. Nat Rev Cancer..

[17] Shigematsu H., Lin L., Takahashi T., Nomura M., Suzuki M., Wistuba I.I., Fong K.M., Lee H., Toyooka S., Shimizu N. (2005). Clinical and biological features associated with epidermal growth factor receptor gene mutations in lung cancers. J. Natl. Cancer Inst..

[18] Manning G., Whyte D.B., Martinez R., Hunter T., Sudarsanam S. (2002). The protein kinase complement of the human genome. Science.

[19] Yasuda H., Kobayashi S., Costa D.B. (2012). *EGFR* exon 20 insertion: Preclinical data and clinical implications. Lancet Oncol..

[20] Giridharan S., Ansari J., Hussain I. (2025). Advancing *EGFR* exon 20 insertion-mutated non-small cell lung cancer (NSCLC) management through molecular diagnostics and targeted therapies. Cureus.

[21] Wen Q., Zhuang Y., Fu S., Pan C., Liu Z., Wang L. (2025). Efficacy and safety of later-line targeted therapies in advanced non-small cell lung cancer with *EGFR* exon 20 insertion mutations: A systematic review. Front Pharmacol..

[22] Piotrowska Z., Tan D.S., Smit E.F., Spira A.I., Soo R.A., Nguyen D., Lee V.H., Yang J.C., Velcheti V., Wrangle J.M. (2023). Safety, tolerability, and antitumor activity of zipalertinib among patients with non-small-cell lung cancer harboring epidermal growth factor receptor exon 20 insertions. J. Clin. Oncol..

[23] Elamin Y.Y., Robichaux J.P., Carter B.W., Altan M., Gibbons D.L., Fossella F.V., Lam V.K., Patel A.B., Negrao M.V., Le X. (2022). Poziotinib for *EGFR* exon 20-mutant NSCLC: Clinical efficacy, resistance mechanisms, and impact of mutation subtypes. J. Thorac. Oncol..

[24] Bhullar K.S., Lagarón N.O., McGowan E.M., Parmar I., Jha A., Hubbard B.P., Rupasinghe H.P.V. (2018). Kinase-targeted cancer therapies: Progress, challenges and future directions. Mol. Cancer.

[25] Zhang X., Gureasko J., Shen K., Cole P.A., Kuriyan J. (2006). An allosteric mechanism for activation of the kinase domain of epidermal growth factor receptor. Cell.

[26] Carey K.D., Garton A.J., Romero M.S., Kahler J., Thomson S., Ross S., Park F., Haley J.D., Gibson N., Sliwkowski M.X. (2006). Kinetic analysis of the epidermal growth factor receptor kinase domain reveals kinetically distinct mechanisms of kinase inhibition. J. Biol. Chem..

[27] Yun C.H., Boggon T.J., Li Y., Woo M.S., Greulich H., Meyerson M., Eck M.J. (2007). Structures of lung cancer-derived *EGFR* mutants and inhibitor complexes: Mechanism of activation and resistance. Cancer Cell.

[28] Wood E.R., Truesdale A.T., McDonald O.B., Yuan D., Hassell A., Dickerson S.H., Ellis B., Pennisi C., Horne E., Lackey K. (2004). A unique structure for epidermal growth factor receptor bound to GW572016 (Lapatinib): Relationships among protein conformation inhibitor off-rate, and receptor activity in tumor cells. Cancer Res..

[29] Remon J., Moran T., Majem M., Reguart N., Dalmau E., Marquez-Medina D., Gascon P. (2014). Acquired resistance to epidermal growth factor receptor tyrosine kinase inhibitors in *EGFR*-mutant non-small cell lung cancer: A new era in oncogenic mutations. Cancer Treat. Rev..

[30] Robichaux J.P., Elamin Y.Y., Tan Z., Carter B.W., Zhang S., Liu S., Li S., He J., Patel A.B., Gibbons D.L. (2018). Mechanisms and clinical activity of an *EGFR* and *HER2* exon 20-selective kinase inhibitor in non-small cell lung cancers with EGFR or HER2 exon 20 mutations. Nat. Med..

[31] Sha C., Lee P.C. (2024). *EGFR*-Targeted Therapies: A Literature Review. J. Clin. Med..

[32] Oxnard G.R., Binder A., Janne P.A. (2013). New targetable oncogenes in non-small-cell lung cancer. J. Clin. Oncol..

[33] Chalmers Z.R., Connelly C.F., Fabrizio D., Gay L., Ali S.M., Ennis R., Schrock A.B., Campbell B., Shlien A., Chmielecki J. (2017). Analysis of 100,000 human cancer genomes reveals the landscape of tumor mutational burden. Genome Med..

[34] Yang J.C., Wang M., Doucet L., Fan Y., Lv D., Sun M., Huang D., Greillier L., Planchard D., Hong Q. (2025). Phase II dose-randomized study of sunvozertinib in platinum-pretreated non-small cell lung cancer with epidermal growth factor receptor exon 20 insertion mutations (WU-KONG1B). J. Clin. Oncol..

[35] Zhou C., Fan Y., Wu L., Han B., Wang M., Chen G., Fang J., Li W., Lu S., Zhang L. Efficacy and safety of furmonertinib at 240 mg in patients with *EGFR* exon 20 insertion-positive NSCLC: Results from the FURVENT phase III trial. Proceedings of the WCLC 2024.

[36] Yu H.A., Zhou C., Ramalingam S.S., Fan Y., Wu L., Han B., Wang M., Chen G., Fang J., Li W. Furmonertinib 240 mg in first-line treatment of *EGFR* exon 20 insertion-positive NSCLC: Updated efficacy and safety from the FURVENT trial. Proceedings of the ASCO 2025.

[37] Cho B.C., Lu S., Felip E., Spira A.I., Girard N., Lee J.S., Lee S.H., Han J.-Y., Kim S.-W., Yang J.C. (2024). Amivantamab plus lazertinib in previously untreated EGFR-mutated advanced NSCLC. N. Engl. J. Med..

[38] Yang G., Xu H., Hu J., Liu R., Hu P., Yang Y., Li W., Hao X., Zhang S., Xu F. (2022). Specific *HER2* exon 20 Gly776 deletion-insertions in non-small cell lung cancer: Structural analysis and sensitivity to HER2-targeted tyrosine kinase inhibitors. Front. Pharmacol..

[39] Shi Y., Au J.S., Thongprasert S., Srinivasan S., Tsai C.M., Khoa M.T., Heeber R.A., Sriuranpong V., Mok T., Ford J. (2014). A prospective, molecular epidemiology study of *EGFR* mutations in Asian patients with advanced non-small-cell lung cancer of adenocarcinoma histology (PIONEER). J. Thorac. Oncol..

[40] Li B.T., Smit E.F., Goto Y., Nakagawa K., Udagawa H., Mazieres J., Nagasaka M., Bazhenova L., Saltos A.N., Felip E. (2022). Trastuzumab deruxtecan in *HER2*-mutant non-small-cell lung cancer. N. Engl. J. Med..

[41] Lindeman N.I., Cagle P.T., Aisner D.L., Arcila M.E., Beasley M.B., Bernicker E.H., Colasacco C., Dacic S., Hirsch F.R., Kerr K. (2018). Updated molecular testing guideline for the selection of lung cancer patients for treatment with targeted tyrosine kinase inhibitors: Guideline from the College of American Pathologists, the International Association for the Study of Lung Cancer, and the Association for Molecular Pathology. Arch Pathol. Lab. Med..

[42] Rolfo C., Mack P.C., Scagliotti G.V., Baas P., Barlesi F., Bivona T.G., Herbst R.S., Mok T.S., Peled N., Pirker R. (2021). Liquid biopsy for advanced NSCLC: A consensus statement from the IASLC. J. Thorac. Oncol..

[43] Westover D., Zugazagoitia J., Cho B.C., Lovly C.M., Paz-Ares L. (2018). Mechanisms of acquired resistance to first-and second-generation *EGFR* tyrosine kinase inhibitors. Ann. Oncol..

[44] Le X., Cornelissen R., Garassino M., Clarke J.M., Ali S.M., Goldman J.W., Socinski M.A., Bauml J.M., Levy B., Goto K. (2021). A phase 2 study of poziotinib in *EGFR* or *HER2* exon 20 mutant non-small cell lung cancers. J. Thorac. Oncol..

[45] Park K., Haura E.B., Leighl N.B., Mitchell P., Shu C.A., Girard N., Viteri S., Han J.Y., Kim S.W., Lee C.K. (2021). Amivantamab in post-platinum NSCLC with *EGFR* exon 20 insertions (CHRYSALIS study). J. Clin. Oncol..

[46] Lim S.M., Sabari J.K., Sanborn R.E., Girard N., Zhou C., Park K., Cho B.C. First-line subcutaneous amivantamab plus chemotherapy in *EGFR* exon 20 insertion-mutated advanced NSCLC: Results from PALOMA-2. Proceedings of the IASLC World Conference on Lung Cancer (WCLC) 2025.

[47] Riely G.J., Neal J.W., Camidge D.R., Spira A.I., Piotrowska Z., Costa D.B., Tsao A.S., Patel J.D., Gadgeel S.M., Bazhenova L. (2021). Activity and safety of mobocertinib (TAK-788) in previously treated non–small cell lung cancer with *EGFR* exon 20 insertion mutations from a phase I/II trial. Cancer Discov..

[48] Zhou C., Ramalingam S.S., Kim T.M., Kim S.W., Yang J.C., Riely G.J., Mekhail T., Nguyen D., Garcia Campelo M.R., Felip E. (2021). Treatment outcomes and safety of mobocertinib in platinum-pretreated patients with *EGFR* exon 20 insertion-positive metastatic NSCLC. JAMA Oncol..

[49] Shi Y., Chen G., Wang X., Liu Y., Wu L., Hao Y., Liu C., Zhu S., Zhang X., Li Y. (2022). Furmonertinib (AST2818) versus gefitinib as first-line therapy for Chinese patients with locally advanced or metastatic *EGFR* mutation-positive non-small-cell lung cancer (FURLONG): A multicentre, double-blind, randomised phase 3 study. Lancet Respir. Med..

[50] Hu S., Ming H., He Q., Ding M., Ding H., Li C. (2024). A study of high dose furmonertinib in *EGFR* exon 20 insertion mutation-positive advanced non-small cell lung cancer. Front. Oncol..

[51] Passaro A., Janne P.A., Mok T., Peters S. (2021). Overcoming therapy resistance in *EGFR*-mutant lung cancer. Nat. Cancer.

[52] Piotrowska Z., Isozaki H., Lennerz J.K., Gainor J.F., Lennes I.T., Zhu V.W., Marcoux N., Banwait M.K., Digumarthy S.R., Su W. (2018). Landscape of acquired resistance to osimertinib in *EGFR*-mutant NSCLC and clinical validation of combined EGFR and RET inhibition. Cancer Discov..

